# Supplementary material for: Phenotypic and Safety Assessment of the Cheese Strain Lactiplantibacillus plantarum LL441, and Sequence Analysis of its Complete Genome and Plasmidome
Source: Int J Mol Sci. 2022 Dec 29;24(1):605. doi: 10.3390/ijms24010605 (PMC9820265; doi:10.3390/ijms24010605)
Supplement: Supplementary file 1 [file ijms-24-00605-s001.zip › ijms-2074113-supplementary.pdf]

Table S1. Presence of catabolic genes in the genome (chromosome or plasmids) of *Lactiplantibacillus plantarum* LL441 involved in growth in milk and formation of flavour (taste and aroma) compounds.

Protein/Description	Gene/Operon	Function	Chromosome	Plasmid
**Proteases**				
ATP-dependent zinc metalloprotease	*ftsH*	Cleavage of transmembrane sequences, cell division	1	
Clp protease	*clpCEPX*	Turnover of cellular proteins	1	
ClpCP protease substrate adapter protein	*mecA*	Activates protein degradation by ClpCP complex	1	
DegP/HtrA serine protease	*htrA*	Clearance of denatured or aggregated proteins from the inner-membrane and periplasmic space	1	
Hsl protease	*hslUV*	ATP-binding and protease subunits	1	
Intracellular protease	-	-	1	
Late competence processing protease	*comC*	Processing of DNA during uptake		
Lon-like protease with PDZ domain	-	Quality-control of proteins	1	
Membrane-associated zinc metalloprotease	*yugP*	Hydrolyses Met if followed by Ala, Pro, or Ser	1	
Membrane protease family protein	-	-	1	
Periplasmic protease	-	-	1	
Phage proteases	-	-	1	
SOS-response repressor and protease	*lexA*	Autoproteolysis in SOS response	1	
YmfH protease	*ymfH*		1	
Zinc protease	-	-	1	
**Peptidases**				
D-alanyl-D-alanine dipeptidase	-	-	1	
Aminopeptidase C	*pepC*	General cysteine aminopeptidase	2	
Aminopeptidase YpdF	*ypdF*	Hydrolyses Xaa-Pro bonds when Xaa is Ala, Asn or Met	1	
D-alanyl-D-alanine carboxypeptidase	*dacA*	Cross-linking of peptidoglycan chains; PBP	2	
Dipeptidase	-	-	4	
ImmA/IrrE family metallo-endopeptidase	-	-	-	1 pLL441-1
Lysyl aminopeptidase	-	-	1	
M15 family metallopeptidase	-	Cell wall biosynthesis	1	
Methionine aminopeptidase	*pepM*	Release of Met from proteins and peptides	1	
Multimodular transpeptidase-transglycosylase	-	Peptidoglycan biosynthesis, probably PBPs	2	
Muramoyl tetrapeptide carboxypeptidase	-	-	1	

Neutral endopeptidase	*pepO*	Degradation of casein-derived peptides	1	
Oligoendopeptidase F	*pepF*	Hydrolyses 7-17 amino acids long peptides	2	
Proline iminopeptidase	*pepI*	Releases proline from peptides	3	
SprT-family zinc metallopeptidase	*sprT*	-	1	
Tripeptide aminopeptidase	*pepT*	Amino degradation of tripeptides	1	
Xaa-His dipeptidase	*pepV*	Hydrolysis of Xaa-His dipeptides	1	
Xaa-Pro dipeptidase	*pepX*	Release dipeptides when Pro is at the second position	2	
**Lactose, citrate, amino acid, and peptide transporters**				
Amino acid permease, GabP family	-	Transport of amino acids	5	
Amino acid permease, YdaO	*ydaO*	Transport of amino acids	1	
Cysteine ABC transporter	*cydCD*	Transport of cysteine	1	
Di-tripeptide/cation symporter DtpT	*dtpT*	Transport of di- and tripeptides	1	
D-serine/D-alanine/glycine transporter	-	Transporter of serine, alanine, glycine	3	
Glutamine ABC transporter	*glnHPQ*	Transport of basic amino acids	1	
Lactose permease	*lacP*	Transport of lactose	2	1, pLL441-2
β-galactosidase	*lacZ*	Lactose degradation	1	
β-galactosidase LacLM	*lacLM*	Lactose degradation	1	1, pLL441-2
Methionine ABC transporter	-	Transport of Met	3	
Na(+)-dependent branched-chain amino acid transporter	*brnQ*	Transport of branched chain amino acids	2	
Oligopeptide ABC transporter	*oppABCDF*	Transport of oligopeptides	1	
Oligopeptide ABC transporter	*oppA*	Substrate binding protein	5	
Proton/glutamate symporter	-	Transport of glutamate	1	
Serine transporter	-	Transport of serine	1	
Tyrosine transporter	*nhaC*	Transport of tyrosine	2	
**Aminotransferases and transaminases**				
Aspartate aminotransferase	*aspAT*	Reversible transfer of a α-amino group between Asp and Glu	6	
Branched-chain amino acid aminotransferase	*bcaT*	Cys, Val, Leu, Iso and Met, synthesis and metabolism, synthesis of secondary metabolites	1	
Glutamine amidotransferase, class 1	*pfpI*	Removal of the ammonia group from glutamine	2	
Glutamine-dependent 2-keto-4-methylthiobutyrate transaminase	-	Cys and Met metabolism, metabolic pathways	1	
Glutamine-fructose-6-P aminotransferase	*glmS*	Ala and Glu, metabolism, metabolic pathways	1	
Histidinol-phosphate aminotransferase	-	Glu and His metabolic pathways	1	

L,D-transpeptidase	-	Cross-linking of peptide stems in peptidoglycans	1	
Multimodular transpeptidase-transglycosidase	-	-	2	
N-acetylornithine aminotransferase	*rocD*	Arg biosynthesis, metabolic pathways, synthesis of secondary metabolites	1	
Phosphoserine aminotransferase	-	Gly, Ser, Thr, Cys and Met metabolism, synthesis of secondary metabolites	1	
Pyruvate transaminase	-	Ala metabolism	1	
**Dehydrogenases**				
1,3-propanediol dehydrogenase	-	Glycerolipid, propanoate metabolism	1	
Aldehyde dehydrogenase	*adhE*	Fatty acids degradation, amino acids synthesis and degradation, synthesis of secondary metabolites	1	
Alanine dehydrogenase	*ala-dh*	Ala, Asp and Glu metabolism, metabolic pathways	1	
Alcohol dehydrogenase class III	*adh*	Glycolysis, fatty acid degradation, Tyr metabolism, pyruvate metabolism	3	1, pLL441-2
D-lactate dehydrogenase	*ldhD*	Synthesis of secondary metabolites	2	
L-lactate dehydrogenase	*ldhL*	Glycolysis, Cys and Met metabolism, synthesis of secondary metabolites	5	
Homoserine dehydrogenase	-	Gly, Ser, Thr and Cys, Met metabolism, Lys synthesis, metabolic pathways	2	
NADP-specific glutamate dehydrogenase	*gdhA*	Arg synthesis, Ala and Asp metabolism, metabolic pathways	1	
Histidinol dehydrogenase	-	His biosynthesis and biosynthesis of secondary metabolites	1	
**Lyases**				
Argininosuccinate lyase	-	Arg biosynthesis, Ala, Asp and Glu metabolism, metabolic pathways, biosynthesis of secondary metabolites	1	
Argininosuccinate synthase	-	Arg biosynthesis, Ala, Asp and Glu metabolism, metabolic pathways, biosynthesis of secondary metabolites	1	
Citrate lyase complex	*citCDEFG*	Formation of pyruvate from citrate	1	
Cystathionine beta-lyase	*cglB*	Production of sulphur compounds	1	
Cystathionine beta-synthase	*cbs*	Transulfuration from homocysteine to cystathionine	1	
Cystathionine gamma-lyase	*cglA*	Gly, Ser, Met, Cys and Thr metabolism, metabolic pathways	1	
Cystathionine gamma-synthase	*cgs*	Formation of cystathionine from cysteine	1	
D-serine ammonia-lyase	-	Gly, Ser and Thr metabolism, D-amino acid metabolism, metabolic pathways	1	
S-ribosylhomocysteine lyase	*luxS*	Cys and Met metabolism, metabolic pathways	1	

Aspartate ammonia-lyase	*aspA*	Ala, Asp and Glu metabolism	1
Aspartate ammonia-ligase	*asnA*	Ala, Gsp and Glu metabolism	1
**Esterases/lipases**			
Alpha/beta hydrolase	-	Lipid transport and metabolism	3
Hydrolase HAD	-	-	7
Esterase/lipase	*estA*	Lipid transport and metabolism	1
Glycerophosphoryl diester phosphodiesterase	*glpQ*	Glycerophospholipid metabolism	2
Lipase/Acylhydrolase	-	-	1
Phosphoesterase	-	Release of membrane-linked proteins with glycosylphosphatidylinositol anchors	1

Colours denote different functional categories.

Table S2. Presence of "probiotic marker genes" (PMGs[a]) involved in stress resistance (acid, osmotic, oxidative, temperature), bile salt hydrolase activity, adhesion ability or gut persistence in the *Lactiplantibacillus plantarum* LL441 genome (chromosome or plasmids).

**Protein/description**	**Gene/operon**	**Function**	**Chromosome**	**Plasmid**
1-acylglycerol-3-phosphate *O*-acyltransferase	*plsC*	Acid stress	1	
60 kDa chaperonin	*groL* / *groEL*	Acid stress	1	
ATP synthase	*atpABCDEFGH*	Acid stress	1	
ATP-dependent Clp protease	*clpCEPX*	Acid stress	1	
Chaperone protein ClpB	*clpB*	Acid stress	1	
D-alanine-D-alanyl carrier protein ligase	*dltA*	Acid stress	1	
Glucose-6-phosphate isomerase	*gpi*	Acid stress	1	
Glutamate decarboxylase	*gadB*	Acid stress	1	
Glyceraldehyde-3-phosphate dehydrogenase	*gap*	Acid stress	1	
GMP synthase [glutamine-hydrolyzing]	*guaA*	Acid stress	1	
GTP pyrophosphokinase	*relA*	Acid stress	2	
L-lactate dehydrogenase	*ldh*	Acid stress	5	
D-alanine transfer protein	*dltD*	Acid stress	1	
Protein RecA	*recA*	Acid stress	1	
Pyruvate kinase	*pyk*	Acid stress	1	
Triosephosphate isomerase	*tpiA*	Acid stress	1	
Chaperone protein DnaK / HSP-70	*dnaK*	Acid stress / bile resistance	1	
Copper transporting ATPase	*copA*	Acid stress / bile resistance	1	-
Enolase	*eno*	Acid stress / bile resistance	2	
Ornithine carbamoyltransferase	*arcB*	Acid stress / bile resistance	1	
Phosphoglycerate kinase	*pgk*	Acid stress / bile resistance	1	
10 kDa chaperonin	*groS* / *groES*	Acid stress / bile resistance / temperature	1	
Chaperone protein DnaJ	*dnaJ*	Acid stress / bile resistance / temperature	1	
Heat shock protein GrpE / HSP-70 cofactor	*grpE*	Acid stress / bile resistance / temperature	1	
Serine protease HtrA	*htrA* / *degP*	Acid stress / bile resistance / temperature	1	
S-ribosylhomocysteine lyase	*luxS*	Acid stress / bile resistance / temperature / production of antimicrobial compounds	1	
30S ribosomal protein S3	*rpsC*	Bile resistance	1	
30S ribosomal protein S5	*rpsE*	Bile resistance	1	

50S ribosomal protein L4	*rplD*	Bile resistance	1	
50S ribosomal protein L5	*rplE*	Bile resistance	1	
50S ribosomal protein L6	*rplF*	Bile resistance	1	
Arginine–tRNA synthetase (ligase)	*argS*	Bile resistance	1	-
Choloylglycine hydrolase	*cbh / bsh*	Bile resistance	3	
CTP synthase	*pyrG*	Bile resistance	1	
DD-transpeptidase / penicillin-sensitive transpeptidase / penicillin-binding protein 1A	*ponA / pbp1A*	Bile resistance	1	
Dihydrolipoyl dehydrogenase	*pdhD / lpdA*	Bile resistance	1	
DNA protection during starvation protein	*dps*	Bile resistance	1	pLL441-7
Endopeptidase PepO	*pepO*	Bile resistance	1	
Glucosamine-6-phosphate deaminase	*nagB / gnp*	Bile resistance	1	
Glutamine synthetase		Bile resistance	1	
Oligopeptide–binding protein OppA	*oppA*	Bile resistance	6	
Sortase A	*srtA*	Bile resistance / adhesion	1	
Glycerol facilitator–aquaporin Z	*gla*	Osmotic stress	1	
Glycerol uptake facilitator	*glpF*	Osmotic stress	5	
Glycine betaine transport system	*opuAA, opuAB*	Osmotic stress	1	
Choline transport system	*opuBA, opuBB, opuBC, opuBD*	Osmotic stress	1	
Glycine betaine/carnitine transport permease protein GbuB	*gbuB*	Osmotic stress	1	
Peptide methionine sulfoxide reductase MsrB	*msrB*	Oxidative stress	1	
Exodeoxyribonuclease III	*exoA*	Adhesion	1	
Lipoprotein signal peptidase	*lspA*	Adhesion	1	
PTS system, cellobiose-specific EIIC component	*celB*	Gut persistance	7	
Pyruvate carboxylase	*pycA*	Adhesion	1	
Trehalose-6-phosphate hydrolase	*treA*	Gut persistance	2	

[a]PMGs as defined by Carpi et al. (2022). Only complete genes are being reported; Colours denote different functional categories.

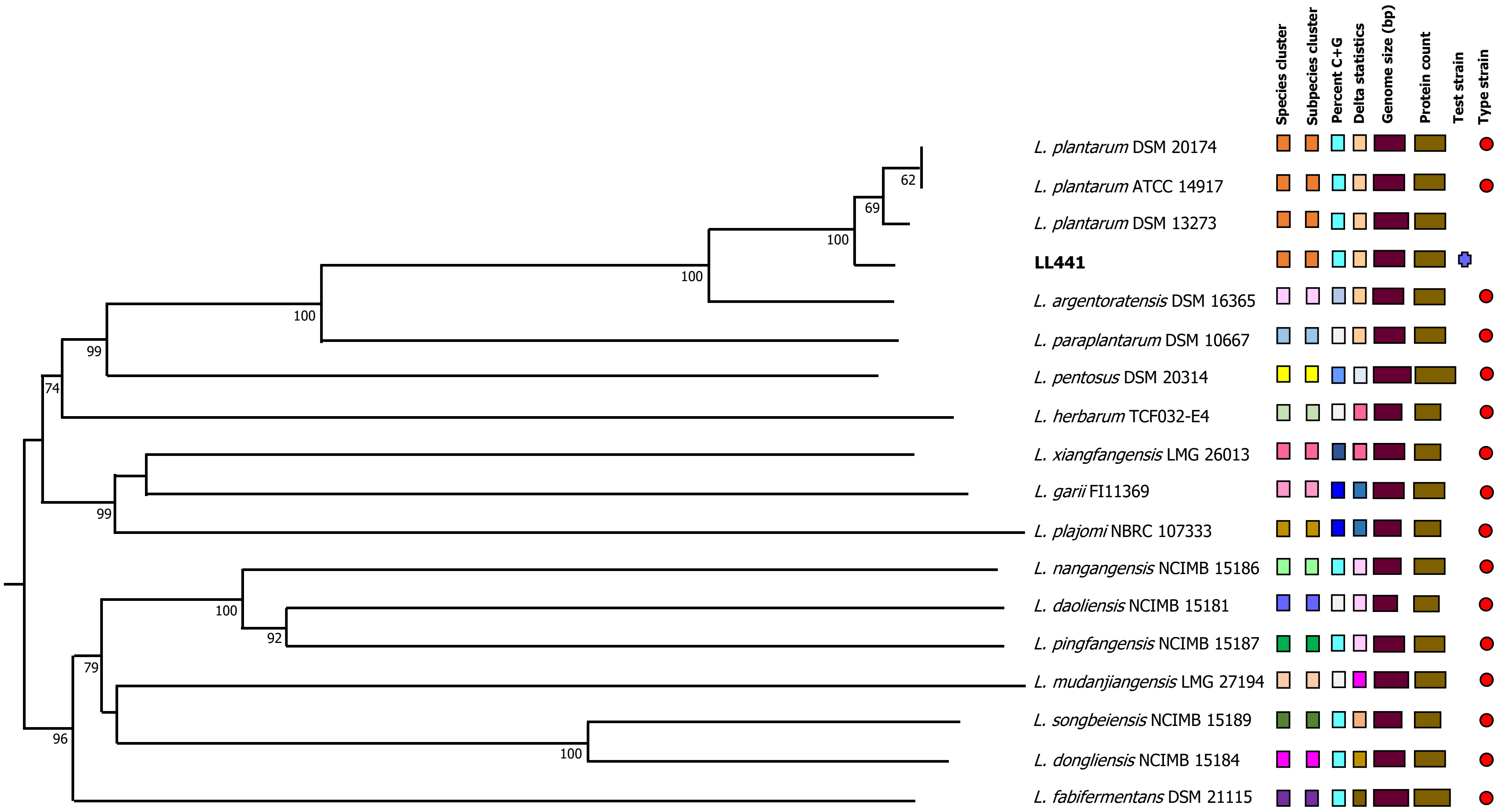

Figure S1.- Phylogenomic analysis of LL441 and strains of species and subspecies of the *Lactiplantibacillus plantarum* group. The tree was inferred from Genome BLAST Distance Phylogeny (GBDP)with FastME 2.1.6.1 at the DSMZ Type Strain Genome Server (http://ggdc.dsmz.de/; Accessed on November 2022). Branch lengths were scaled in terms of the approach distance of formula d5. The numbers above branches are GBDP pseudo-bootstrap support values >60% from 100 replications (average branch support of 85.2%). The tree was rooted at the midpoint. The colour scheme on the right denotes with the same tone strains considered as belonging to the same taxon.
